# Activation of the transcription factor carbohydrate-responsive element-binding protein by glucose leads to increased pancreatic beta cell differentiation in rats

**DOI:** 10.1007/s00125-012-2623-0

**Published:** 2012-07-05

**Authors:** A. Soggia, K. Flosseau, P. Ravassard, G. Szinnai, R. Scharfmann, G. Guillemain

**Affiliations:** 1 INSERM U845, Research Center Growth and Signalling, Université Paris Descartes, Sorbonne Paris Cité, Faculté de Médecine, Hôpital Necker, Paris, France; 2 CNRS – UMR 7225, CNRS – UMR 7225 Hôpital Pitié Salpêtrière, Paris, France; 3 Department of Biomedicine, University of Basel, Basel, Switzerland

**Keywords:** Carbohydrate-responsive element-binding protein, Glucose metabolism, Insulin, Pancreatic beta cell differentiation, Pancreatic development, Pentose phosphate pathway

## Abstract

**Aims/hypothesis:**

Pancreatic cell development is a tightly controlled process. Although information is available regarding the mesodermal signals that control pancreatic development, little is known about the role of environmental factors such as nutrients, including glucose, on pancreatic development. We previously showed that glucose and its metabolism through the hexosamine biosynthesis pathway (HBP) promote pancreatic endocrine cell differentiation. Here, we analysed the role of the transcription factor carbohydrate-responsive element-binding protein (ChREBP) in this process. This transcription factor is activated by glucose, and has been recently described as a target of the HBP.

**Methods:**

We used an in vitro bioassay in which pancreatic endocrine and exocrine cells develop from rat embryonic pancreas in a way that mimics in vivo pancreatic development. Using this model, gain-of-function and loss-of-function experiments were undertaken.

**Results:**

ChREBP was produced in the endocrine lineage during pancreatic development, its abundance increasing with differentiation. When rat embryonic pancreases were cultured in the presence of glucose or xylitol, the production of ChREBP targets was induced. Concomitantly, beta cell differentiation was enhanced. On the other hand, when embryonic pancreases were cultured with inhibitors decreasing ChREBP activity or an adenovirus producing a dominant-negative ChREBP, beta cell differentiation was reduced, indicating that ChREBP activity was necessary for proper beta cell differentiation. Interestingly, adenovirus producing a dominant-negative ChREBP also reduced the positive effect of *N*-acetylglucosamine, a substrate of the HBP acting on beta cell differentiation.

**Conclusions/interpretation:**

Our work supports the idea that glucose, through the transcription factor ChREBP, controls beta cell differentiation from pancreatic progenitors.

**Electronic supplementary material:**

The online version of this article (doi:10.1007/s00125-012-2623-0) contains peer-reviewed but unedited supplementary material, which is available to authorised users.

## Introduction

The mature pancreas contains exocrine acinar cells that secrete digestive enzymes into the intestine, and endocrine islets that synthesise hormones such as insulin (beta cells), glucagon (alpha cells), somatostatin (delta cells) and pancreatic polypeptide (PP cells). The pancreas originates from the dorsal and ventral regions of the foregut endoderm directly posterior to the stomach. The first indication of pancreatic morphogenesis occurs in mice at embryonic day (E) 8.5 (E9.5 in rat), when the endoderm evaginates to form buds. Subsequently, the mesenchyme condenses around the underlying endoderm and the epithelial buds grow in size, while the exocrine and endocrine cells differentiate [1].

During development, the endodermal region committed to form the pancreas initially produces the transcription factor pancreatic and duodenal homeobox 1 (Pdx-1), a marker of pancreatic progenitors also produced in mature beta cells [2]. The basic helix–loop–helix factor neurogenin 3 (Neurog3) is then transiently produced in epithelial pancreatic progenitor cells prior to endocrine differentiation [3]. Neurog3 controls the expression of NeuroD, which is another member of the basic helix–loop–helix transcription factor family [4].

Pancreatic development is controlled by signals derived from tissues in contact with the endodermal region that gives rise to the pancreas [5]. Pancreatic development also depends on environmental signals such as oxygen tension [6, 7] and nutrients [8]. We previously showed that glucose controls beta cell development [9]. Specifically, we found that glucose favours pancreatic endocrine cell development by regulating the transition between Neurog3 and NeuroD [9].

Typically, glucose is transported inside the cell and is phosphorylated into glucose 6-phosphate. It next enters three pathways: the glycolysis pathway to provide energy, the hexosamine biosynthetic pathway (HBP) and the pentose phosphate pathway [10]. We previously demonstrated that the positive effect of glucose on beta cell development required its metabolism through the HBP [11]. This pathway produces UDP-*N*-acetyl-glucosamine (UDP-GlcNAc), a substrate for *N*- and *O*-glycosylation, as well as for *O*-GlcNAcylation, which consists of the addition of a single GlcNAc moiety to serine and threonine residues (for a review, see Bouche et al [12]). This last modification is highly dynamic and often compared with phosphorylation.

Interestingly, the pentose phosphate pathway is an alternative pathway for glucose metabolism that generates NADPH and synthesises pentose sugars [13]. Xylulose 5-phosphate, an intermediate of the pentose phosphate pathway, activates type 2A protein phosphatase (PP2A), which in turn dephosphorylates and activates transcription factor carbohydrate-responsive element binding protein (ChREBP) [14, 15]. ChREBP is a transcription factor that belongs to the basic helix–loop–helix leucine zipper family, which transactivates glucose-responsive genes such as those encoding acetyl-CoA carboxylase (ACCase) and liver-type pyruvate kinase (L-PK) by binding the carbohydrate-responsive element [16]. To the best of our knowledge, little information is available concerning the role of ChREBP in pancreatic cell development.

Here, we tested the possible involvement of ChREBP in pancreatic development, and the role of ChREBP as a mediator of glucose effect on beta cell differentiation. For this purpose, we modulated ChREBP activity in an in vitro model of pancreatic development and analysed beta cell differentiation.

## Methods

### Animals and dissection of dorsal pancreatic rudiments

Pregnant Wistar rats were purchased from CERJ (Le Genest, St Isle, France). The first day post-coitum was designated embryonic day 0.5 (E0.5). Pregnant rats at 13.5 days of gestation were killed by carbon dioxide asphyxiation in compliance with the French Animal Care Committee’s guidelines. Dorsal pancreatic buds from E13.5 rat embryos were dissected as previously described [17]. Pancreases from E18.5 WT or *Neurog3* ^–/–^ mice were dissected and used for RNA extraction.

### Organ culture, PP2A inhibitor treatments and BrdU incorporation

Pancreases were laid on 0.45 μm filters (Millipore, St-Quentin-en-Yvelines, France) at the air–medium interface in Petri dishes containing RPMI 1640 (Lonza, Basel, Switzerland) supplemented with penicillin (100 U/ml), streptomycin (100 μg/ml), HEPES (10 mmol/l), non-essential amino acids (1X; Invitrogen, Cergy-Pontoise, France) and 10% heat-inactivated FCS (HyClone, Logan, UT, USA) (complete RPMI culture medium) [18]. Cultures were maintained at 37°C in humidified 95% air–5% CO_2_. d-Glucose, xylitol, okadaic acid, calyculin A and GlcNAc (Sigma-Aldrich, Lyon, France) were used at the indicated concentrations. It has to be noted that, in the medium without added glucose, about 1 mmol/l glucose is added by the serum. The medium was changed every other day. For cell proliferation assays, 10 μmol/l BrdU (Sigma-Aldrich) was added to the medium during the last hour of culture. At the end of the indicated culture period, the pancreatic rudiments were photographed.

### Pancreatic dissociation and adenoviral infection

Pancreases were dissected and kept in PBS–2% FCS, before being resuspended in 500 μl Hank’s balanced salt solution (HBSS) containing collagenase V (1 mg/ml) and DNase (20 μg/ml) (both from Sigma-Aldrich). The dissociation was performed at 37°C in a thermomixer (Eppendorf, Le Pecq, France). At the end of the dissociation, complete RPMI culture medium was added to the cell suspension which was then centrifuged for 1 min at 2,000 *g*. The cells were washed three times and divided into aliquots in 1.5 ml tubes (5 × 10^4^ cells/tube) in 500 μl complete RPMI culture medium supplemented with ROCK inhibitor (7 μg/ml; Sigma-Aldrich). The tubes were centrifuged for 1 min at 2,000 *g* and kept open overnight in the incubator. During this period, cells reaggregated and formed clusters that were transferred on a filter and cultured for 6 further days in complete RPMI culture medium supplemented with ROCK inhibitor. For adenoviral infection, dissociated cells were cultured in 50 μl RPMI 1640 with adenoviruses (multiplicity of infection of two) producing a dominant-negative *ChREBP* (also known as *Mlxipl*) into the basic region of the DNA binding domain (dn*ChREBP*) [19], or green fluorescent protein (GFP) as a control for 2 h 30 min at 37°C. At the end of the infection period, 500 μl complete RPMI medium was added, and cells were centrifuged for 1 min at 3,000 rpm and reaggregated as described above, before being transferred on a floating filter for 6 days.

### Immunohistochemistry

Tissues were fixed in 10% formalin, pre-embedded in low-gelling agarose, and embedded in paraffin. All sections (4 μm thick) of each pancreatic explant were collected and processed for immunohistochemical analysis as previously described [20, 21]. Antibodies were used in the following dilutions: mouse anti-insulin, 1/2,000; rabbit anti-insulin, 1/2,000; rabbit anti-amylase, 1/300 (all from Sigma-Aldrich); rabbit anti-proconvertase 1/3 (PC1/3), 1/1,000 (gift from Dr Steiner, University of Chicago, IL, USA); mouse anti-synaptophysin, 1/10 (Dako, Trappes, France); rabbit anti-PDX1 T1/1,000 T[22]; mouse anti-GFP, 1/500 (Euromedex, Souffelweyersheim, France); mouse anti-BrdU, 1/4 (Amersham Biosciences, Amersham, Buckinghamshire, UK). The fluorescent secondary antibodies used included fluorescein anti-rabbit and Texas red anti-mouse antibodies (both from Jackson ImmunoResearch, Newmarket, Suffolk, UK) and Alexa Fluor 488 anti-rabbit antibody (Invitrogen). Nuclei were stained with Hoechst 33342 (0.3 μg/ml; Invitrogen).

TUNEL experiments were performed using an in situ cell death detection kit (Roche, Neuilly-sur-Seine, France), following the manufacturer’s instructions, on pancreases cultured for 7 days in the presence of glucose 10 mmol/l or xylitol 10 mmol/l alone or supplemented with okadaic acid or calyculin A at the indicated concentrations. Results are expressed as the percentage of apoptotic cells per total number of pancreatic cells, determined by the quantification of nuclei stained in blue with Hoechst 33342.

### Quantification

All sections from each pancreatic rudiment were digitised using cooled, three-charge-coupled-device cameras (Hamamatsu, Middlesex, NJ, USA) linked to a fluorescence microscope (Leitz DMRB; Leica, Rueil-Malmaison, France). On every image, the surface area of each staining was quantified using ImageJ 1.34 s and summed to obtain the total surface area per explant in mm^2^ as previously described [9, 23]. To quantify the proliferation of early PDX1-positive pancreatic progenitors, we counted the frequency of BrdU-positive nuclei among 2,000 early PDX1-positive progenitors per rudiment. A similar analysis was performed in order to quantify the proliferation of insulin-positive cells. In order to determine the rate of cell proliferation in pancreases cultured in the presence of glucose or xylitol alone, or supplemented with okadaic acid or calyculin A, we determined the percentage of BrdU-positive cells among all pancreatic cells. At least four explants grown under each set of culture conditions were analysed. Results are expressed as means ± SEM.

### RNA extraction and real-time PCR

Total RNA was extracted from pools of three pancreases using an RNeasy Microkit (Qiagen, Courtaboeuf, France) and reverse-transcribed using SuperScript reagents (Invitrogen). RT-PCR was performed using the 7300 Fast RT-PCR system (Applied Biosystems, Courtaboeuf, France). Oligonucleotide sequences are available upon request. Peptidylpropyl isomerase A was used as endogenous control, and E16.5 pancreas cDNA was used as calibrator sample. Data were analysed by the comparative CT method and presented as the fold change in gene expression normalised to an E16.5 calibrator that equalled a value of 1 [9]. At least three pools of explants were analysed per condition, and the results are expressed as means ± SEM.

### Statistics

Results are expressed as means ± SEM. Statistical significance was determined using the Student’s *t* test when only two sets of data were compared. For larger analyses, a non-parametric Kurskal and Wallis test was performed, followed by a Mann–Whitney *U* test.

## Results

### Expression of the transcription factor ChREBP during pancreatic development

**Fig. 1:**
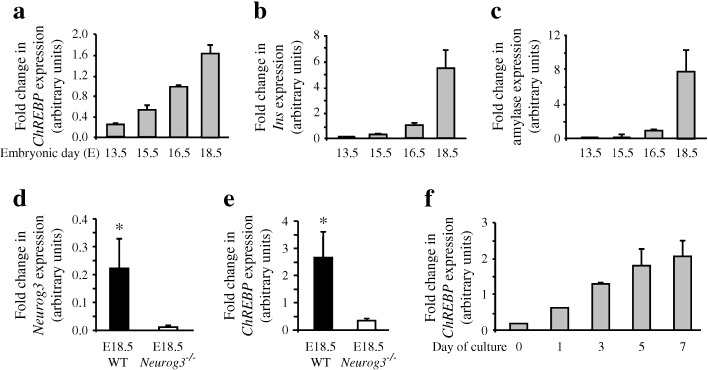
Levels of the transcription factor ChREBP during pancreatic development. RT-PCR quantification of *ChREBP* (**a**), *Ins* (**b**) and amylase (**c**) mRNA in the pancreas at various stages of embryonic development (E13.5, E15.5, E16.5 and E18.5). (**d**,**e**) RT-PCR quantification of *Neurog3* and *ChREBP* mRNA in E18.5 pancreases of wild type or *Neurog3* ^–/–^ mice. (**f**) RT-PCR quantification of *ChREBP* mRNA in E13.5 rat pancreases before (day 0) and after 1, 3, 5 and 7 days of culture. All values are means ± SEM of minimum three independent experiments. *p < 0.05

We first analysed the *ChREBP* expression pattern during pancreatic development. *ChREBP* mRNA was detected in vivo in the pancreas as early as E13.5, its expression gradually increasing at E15.5, E16.5 and E18.5, following a pattern that resembled the one found for *Ins* and amylase mRNA (Fig. 1a–c). ChREBP levels decreased sharply in pancreases from *Neurog3*-deficient mice, indicating that its expression was highly enriched in the endocrine pathway (Fig. 1d). In an in vitro model of pancreatic development in which acinar and endocrine cells developed from E13.5 pancreases [18], *ChREBP* expression level increased as cell differentiation occurred (Fig. 1f).

### Xylitol and glucose treatments induce the expression of ACCase (also known as Acaca) and L-PK (also known as Pklr), two direct targets of ChREBP

**Fig. 2:**
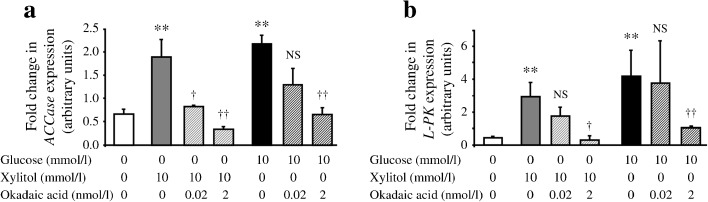
Effect of glucose, xylitol or okadaic acid treatment on the expression of *ACCase* and *L-PK*, two ChREBP targets. (**a**,**b**) Quantification by RT-PCR of *ACCase* (**a**) and *L-PK* (**b**) mRNAs after 7 days of culture of E13.5 rat pancreases in the presence or absence of xylitol (10 mmol/l), glucose (10 mmol/l) and okadaic acid (2 or 0.02 nmol/l). Values are means ± SEM of three independent experiments. **p < 0.01 compared with pancreases cultured without glucose. ^†^ *p* < 0.05 and ^††^ *p* < 0.01 when compared with pancreases cultured without okadaic acid

We first tested the effect of xylitol and glucose on the expression of *ACCase* and *L-PK* mRNA, two direct targets of ChREBP. Xylulose 5-phosphate, an intermediate of the non-oxidative branch of the pentose phosphate pathway, activates PP2A, which will dephosphorylate and activate ChREBP [24]. Treatment with either glucose or xylitol, a precursor of xylulose 5-phosphate, can thus activate ChREBP [25]. In E13.5 rat pancreases in culture, both xylitol and glucose induced *ACCase* and *L-PK* mRNA levels (Fig. 2). This induction occurred in a glucose dose-dependent manner (see electronic supplementary material [ESM] Fig. 1).

Treatment with okadaic acid, a PP2A inhibitor, reversed xylitol-induced activation of *ACCase* and *L-PK* expression, an effect that was stronger at 2 nmol/l than at 0.02 nmol/l (Fig. 2). Such an effect was also observed with calyculin A, another PP2A inhibitor (data not shown). Interestingly, okadaic acid treatment also reversed glucose-induced activation of *ACCase* and *L-PK* expression, but the effect was observed only with 2 nmol/l okadaic acid (Fig. 2).

### Glucose and xylitol treatments activate beta cell development

**Fig. 3:**
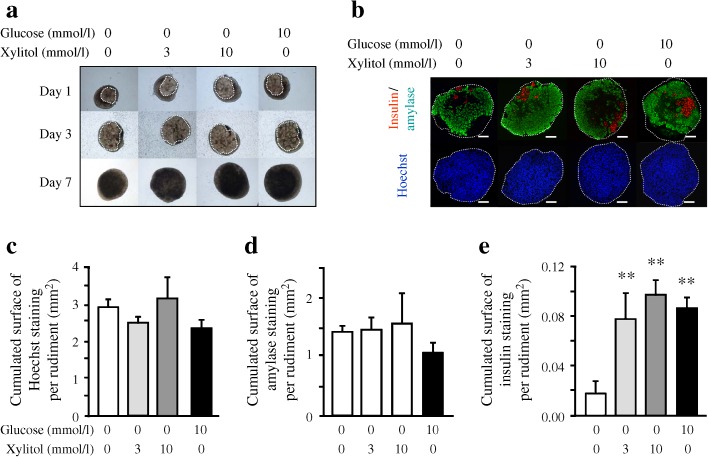
Glucose and xylitol activate the development of insulin-positive cells. (**a**) E13.5 rat pancreases were cultured for 7 days in the presence or absence of xylitol (3 or 10 mmol/l) or glucose (10 mmol/l). Representative images after 1, 3 and 7 days in culture are shown. Pancreatic epithelium is circled in white. (**b**) Immunohistological analysis of pancreases after 7 days of culture in the absence or in presence of xylitol (3 or 10 mmol/l) or in the presence of glucose (10 mmol/l). Insulin was revealed in red and amylase in green. Nuclei are depicted in blue by Hoechst staining. The pancreatic bud is outlined in white. Scale bar: 100 μm. (**c–e**) Quantification of the absolute surface areas occupied by Hoechst-, amylase- and insulin-positive cells after 7 days of culture in the absence or presence of xylitol or glucose

As we observed that the expression of ChREBP targets was induced in response to either glucose or xylitol, we analysed their effects on pancreatic development. To this end, E13.5 rat pancreases were cultured for 7 days in medium without added glucose (control medium) or with glucose (10 mmol/l) or xylitol (3 and 10 mmol/l). Xylitol treatment modified neither pancreatic morphology nor the size of the tissue as quantified following Hoechst staining, nor acinar cell development measured after amylase staining (Fig. 3). However, xylitol increased the development of insulin-producing beta cells, as was the case for glucose, as measured after insulin immunostaining (Fig. 3b–e). This increase in insulin level was observed at both 3 and 10 mmol/l xylitol, and 10 mmol/l xylitol was chosen for subsequent experiments.

Insulin-positive cells that developed in the presence of xylitol produced beta cell markers such as the transcription factor PDX1, an insulin gene transactivator, the pro-convertase 1/3, implicated in proinsulin processing, and synaptophysin, a protein present on the endocrine pancreatic secretory granule membrane (ESM Fig. 2). Such results support the fact that insulin-positive cells that developed in the presence of xylitol are indeed beta cells.

### Glucose and xylitol treatments do not affect pancreatic cell proliferation

Our data indicate that xylitol treatment increased beta cell development. Pancreatic endocrine cells derive from early proliferating pancreatic progenitors producing the transcription factor PDX1 [26, 27]. We first asked whether xylitol increased beta cell mass by acting on the proliferation of either PDX1-positive pancreatic progenitors or mature beta cells. E13.5 pancreases were cultured for 1 or 7 days, BrdU being added during the last hour of culture. In control conditions, 28.45 ± 0.57% of PDX1-positive cells and 2.39 ± 0.35% of insulin-positive cells incorporated BrdU after 1 and 7 days of culture, respectively (ESM Fig. 3). Thus, neither xylitol nor glucose treatment modified rates of proliferation.

### Glucose and xylitol treatments promote beta cell differentiation by activating the transition between Neurog3 and NeuroD

**Fig. 4:**
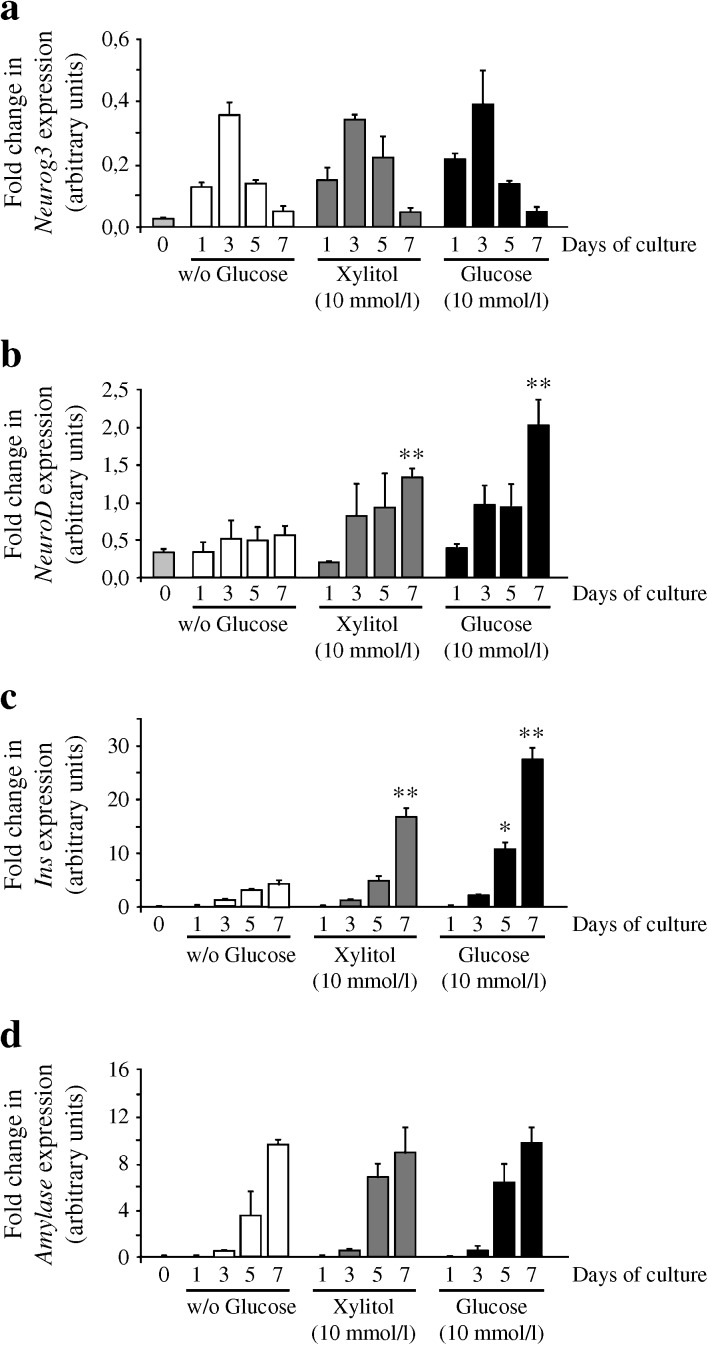
Glucose and xylitol treatments promote beta cell differentiation by activating the transition between NEUROG3 and NeuroD. RT-PCR quantification of *Neurog3*, *NeuroD*, *Ins* and amylase mRNA in E13.5 pancreases before (day 0) and after 1, 3, 5 and 7 days of culture in the presence or absence of xylitol (10 mmol/l) or glucose (10 mmol/l). Comparisons were performed at each time point with pancreases cultured without glucose. Values are means ± SEM of a minimum of three independent experiments. *p < 0.05; **p < 0.01

During development, PDX1-positive pancreatic progenitors give rise to NEUROG3-positive endocrine progenitors that will differentiate into beta cells [3, 28]. In control pancreatic explants, as previously observed [9], *Neurog3* mRNA levels measured by RT-PCR increased after 1 day of culture, peaked at day 3 and decreased thereafter (Fig. 4a). Xylitol treatment did not modify the pattern of *Neurog3* expression (Fig. 4a). On the other hand, when compared with control conditions, xylitol treatment increased the level of NeuroD, a target of NEUROG3 necessary for proper beta cell development (Fig. 4b) [29]. As expected based on the immunohistochemical analysis performed on day 7, xylitol treatment increased the *INS* gene expression pattern (Fig. 4c) without modifying amylase gene expression (Fig. 4d). When glucose was used instead of xylitol, similar effects were observed on endocrine (*Neurog3*, *NeuroD*, *Ins*) and acinar (amylase) cell development. Collectively, these results indicate that xylitol, like glucose, acts specifically on endocrine cell development, by controlling the transition between *Neurog3* and *NeuroD*.

### The effect of glucose and xylitol treatments on beta cell development depends on *ChREBP* activation

**Fig. 5:**
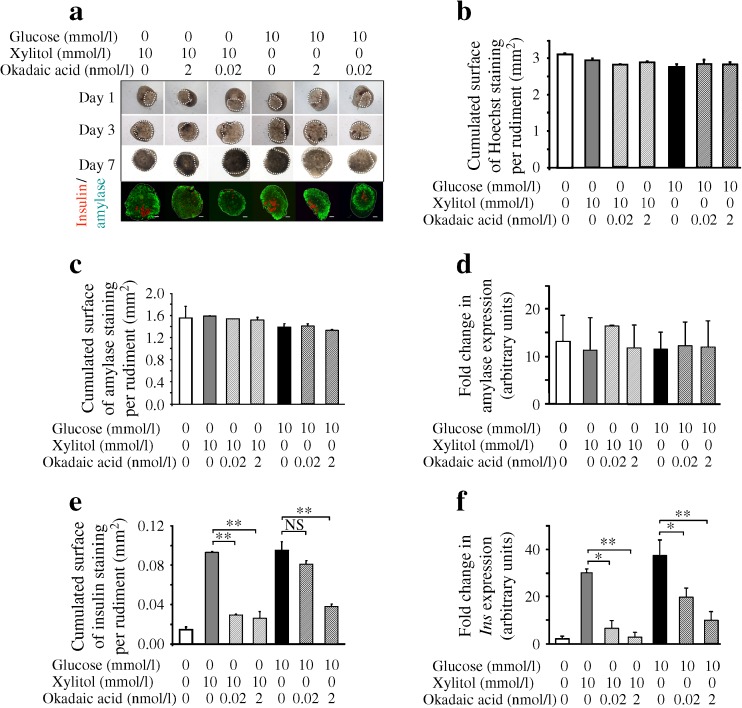
Effect of okadaic acid treatment on the expression of *ACCase* and *L-PK*. (**a**) Representative images of pancreases cultured for 1, 3 and 7 days indicating that okadaic acid treatment did not modify pancreatic shape. Pancreatic epithelium is circled in white. After 7 days of culture, immunohistochemistry experiments were performed on the pancreatic buds. Insulin was revealed in red and amylase in green. Scale bar: 100 μm. (**b**) Quantification of the absolute surface area occupied by Hoechst staining after 7 days of culture of 13.5 rat pancreases in the presence or absence of xylitol (10 mmol/l), glucose (10 mmol/l) or okadaic acid (2 or 0.02 nmol/l). (**c**,**e**) Quantification of the absolute surface area occupied by amylase (**c**) and insulin staining (**e**) after 7 days of culture of 13.5 rat pancreases in the presence or absence of xylitol (10 mmol/l), glucose (10 mmol/l) or okadaic acid (2 or 0.02 nmol/l). (**d**,**f**) Quantification by RT-PCR of amylase (**d**) and *Ins* (**f**) mRNA after 7 days of culture of 13.5 rat pancreases in the presence or in the absence of xylitol (10 mmol/l), glucose (10 mmol/l) and okadaic acid (2 or 0.02 nmol/l). Values are means ± SEM of three independent experiments. *p < 0.05, **p < 0.01 and NS compared with pancreases cultured in the presence of glucose

The data described above indicate that, during pancreatic development, xylitol induces both ChREBP activity and beta cell differentiation. To determine whether the effect of xylitol on beta cell differentiation was dependent on ChREBP activation, we cultured E13.5 pancreases in medium supplemented or not supplemented with xylitol or glucose, in the presence or absence of okadaic acid. Okadaic acid treatment (0.02 or 2 nmol/l) did not modify pancreatic shape and growth (Fig. 5a,b). After 7 days of culture, okadaic acid treatment did not modify acinar cell development quantified by amylase immunostaining and by RT-PCR (Fig. 5a–d). On the other hand, the increase in beta cell development observed with glucose or xylitol treatment was abolished by okadaic acid treatment both at 0.02 and 2 nmol/l, as quantified by insulin immunostaining and by RT-PCR (Fig. 5a–f). A similar effect was observed with calyculin A (data not shown). Importantly, PP2A inhibitors modulate neither cell proliferation nor apoptosis (ESM Fig. 4). An alteration of these variables can therefore not explain the observed decreased in beta cell mass.

To further demonstrate the role of CHREBP in beta cell development, we infected rat embryonic pancreases with an adenovirus producing dnChREBP [19]. For efficient infection, we dissociated E13.5 rat pancreatic cells, infected them with adenoviruses and next reassociated the cells before culture. We first verified that, in this assay, glucose exerted a positive effect on beta cell differentiation, as previously shown with undissociated pancreas [9]. This was indeed the case as glucose induced a 2.56-fold increase in beta cell development without modifying acinar cell development (ESM Fig. 5).

**Fig. 6:**
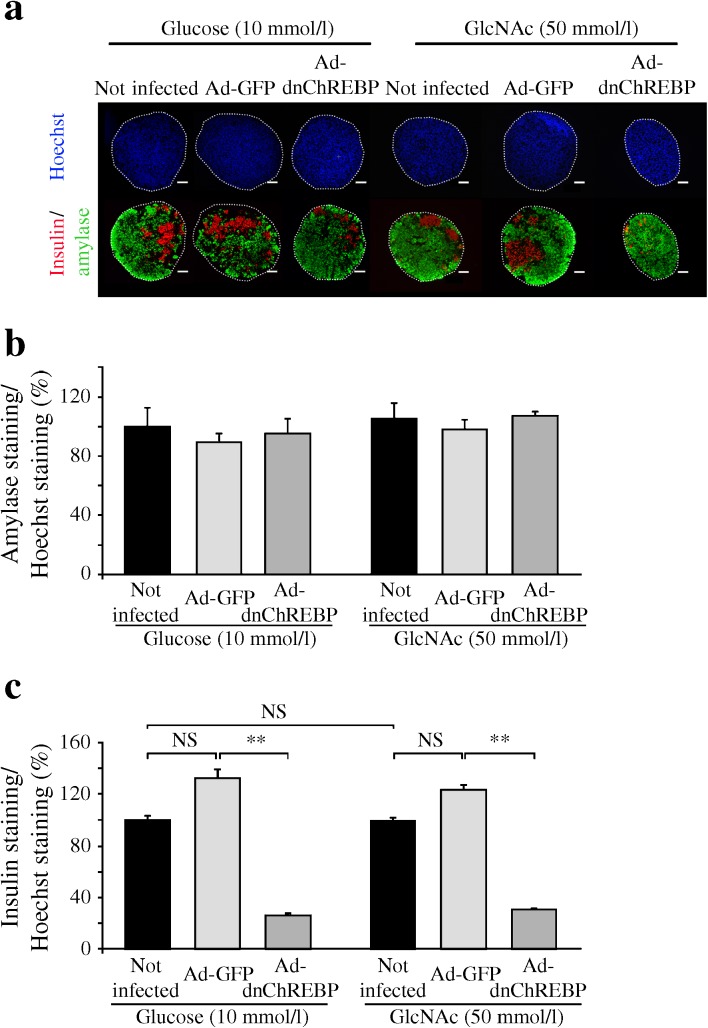
Ad-dnChREBP blocks the effect of glucose and GlcNAc on beta cell development. E13.5 rat pancreases were dissociated, infected or not infected with Ad-GFP or Ad-dnChREBP, reaggregated and cultured in the presence of glucose (10 mmol/l) or in the presence of GlcNAc (50 mmol/l). (**a**) Immunohistological analysis of pancreases after 7 days of culture. Insulin was revealed in red and amylase in green. Nuclei were stained blue using a Hoechst dye. Scale bar: 100 μm. (**b**) Quantification of the relative surface area occupied by amylase-positive cells after 7 days of culture. Values were normalised to the condition ‘not infected and culture in presence of glucose 10 mmol/l’. (**c**) Quantification of the relative surface area occupied by insulin-positive cells after 7 days of culture. Values were normalised to the condition ‘not infected and culture in presence of glucose 10 mmol/l. **p < 0.01

In order to test the efficiency of gene transfer in this model, dissociated cells were infected with an adenovirus producing GFP. Cells were reaggregated, cultured for 2 days, fixed and stained using an anti-GFP antibody. Almost all cells produced GFP, demonstrating that infection was highly efficient (data not shown). We next compared beta and acinar cell development in pancreases that were or were not infected with a GFP or a dnChREBP adenovirus. After 7 days of culture post-infection, the relative mass of amylase-positive cells was similar in all three conditions (Fig. 6a,b). On the other hand, although infection with an adenovirus producing GFP did not modify beta cell development, the relative number of insulin-positive cells was reduced in pancreases infected with an adenovirus producing dnChREBP (Fig. 6a–c). Of note, the percentage of insulin-positive cells that developed in the presence of the adenovirus producing dnChREBP was similar to the one obtained in reaggregated pancreases culture for 7 days in the absence of added glucose (compare Fig. 6c and ESM Fig. 3B).

### The effect of GlcNAc treatment on beta cell development depends on ChREBP activation

We previously demonstrated that the effect of glucose on beta cell development was dependent on its metabolism through the HBP [11]. Specifically, in the absence of added glucose, GlcNAc, a known substrate of the HBP, induced beta cell differentiation from embryonic pancreases and thus reproduced the effects of glucose. We therefore asked whether the effect of GlcNAc treatment on beta cell development was also dependent on ChREBP activation. Dissociated pancreases were infected with an adenovirus producing GFP or dnChREBP, and cultured for 7 days in the absence of glucose but with the addition of GlcNAc 50 mmol/l. We first verified that, in this assay, GlcNAc exerted a positive effect on beta cell differentiation, as previously demonstrated for undissociated pancreases [11]. This was indeed the case as GlcNAc induced beta cell differentiation to the same extent as glucose (Fig. 6c; compare the black columns). Infection with the adenovirus producing GFP did not modify the positive effect of GlcNAc on beta cell differentiation (Fig. 6a,c), whereas when pancreases were infected with the adenovirus producing dnChREBP, beta cell differentiation was reduced (Fig. 6a,c). Again, none of the conditions modified acinar cell differentiation (Fig. 6a,b). These results demonstrated that the positive effect of glucose and GlcNAc on beta cell differentiation require active ChREBP.

## Discussion

Pancreatic beta cell development is a multistep process. Information is now available on the signals that regulate early steps of the process, i.e. the development of PDX1-positive pancreatic progenitors from definitive endoderm [30–32]. On the other hand, less is known about signals controlling beta cell differentiation from PDX1-positive pancreatic progenitors. This step in development can be further divided into three consecutive steps: (1) the proliferation of PDX1-positive pancreatic progenitors; (2) their differentiation into acinar tissue or into NEUROG3-producing endocrine progenitors; and (3) the differentiation of NEUROG3-producing endocrine progenitors into insulin-producing beta cells [33]. Here, by using activators and inhibitors of the pentose phosphate shunt, we demonstrated that this pathway modulated neither the proliferation of PDX1-positive pancreatic progenitors, nor acinar cell differentiation, nor the formation of NEUROG3-producing endocrine progenitors. On the other hand, we demonstrated that the positive effect of glucose and xylitol on pancreatic beta cell differentiation depended on the transcription factor ChREBP and occurred in NEUROG3-producing endocrine progenitors.

In this study, we used an in vitro bioassay that recapitulates the major steps occurring during beta cell development from PDX1-positive fetal pancreatic progenitor cells. In this model, PDX1-positive pancreatic progenitors first proliferate and next differentiate into endocrine or acinar tissue. We previously validated and used this model to define factors and conditions that regulate specific steps in beta cell development. Specifically, with this assay, we previously demonstrated that fibroblast growth factor 10 was an activator of the proliferation of PDX1-positive pancreatic progenitors [18]. Here, we found that xylitol did not regulate cell proliferation during pancreatic development, as previously shown for glucose [9]. We also previously used this model of in vitro pancreatic development to define signals and conditions that modulate the differentiation of PDX1-positive pancreatic progenitors into NEUROG3-positive endocrine progenitors. We demonstrated that small molecules such as histone deacetylase inhibitors [23], branched amino acids [34] and oxygen tension [7] regulate the development of NEUROG3-positive endocrine progenitors from PDX1-positive pancreatic progenitors. Recently, using the same assay, we demonstrated that glucose regulates the next step in beta cell development, i.e. the differentiation of beta cells from NEUROG3-positive endocrine progenitors, and that glucose metabolism was implicated in this regulation at least in part through activation of the HBP [9, 11].

In the present study, we demonstrated that the transcription factor ChREBP regulates beta cell differentiation. ChREBP regulates gene transcription in response to glucose (for a review, see [24]). Specifically, the pentose phosphate pathway, and particularly xylulose 5-phosphate, selectively activates PP2A, which in turn dephosphorylates the transcription factor ChREBP [14]. This allows ChREBP translocation into the nucleus and activation of ChREBP targets, such as the glycolytic gene *L-PK* [35] or the lipogenic genes *ACCase* and *Fas* (which encodes fatty acid synthase) [36]. ChREBP production is most abundant in liver, and is small in kidney and white and brown adipose tissue [16]. In adult islets, ChREBP controls the expression of a number of genes such as *L-PK* [37], *ACCase* [38], *Fas* [39], thioredoxin-interacting protein [40] and aryl hydrocarbon receptor nuclear translocator [41]. Interestingly, recent data suggest that ChREBP can also be activated by glucose 6-phosphate [42] and by the HBP [43, 44], placing ChREBP at the crossroads of all glucose metabolic pathways.

Little information has been available on the role of ChREBP during pancreatic development. Different arguments derived from the present study indicate that ChREBP is important for proper beta cell differentiation. First, as described in this study, ChREBP is expressed in the pancreas during development [45]. Second, during prenatal life, ChREBP is highly enriched in the endocrine pathway, as demonstrated by its sharp decrease in pancreases from *Neurog3*-deficient mice that lack endocrine cells. ChREBP can thus be added to the list of genes whose expression is enriched in pancreatic endocrine cells [46]. Third, in the embryonic pancreas, xylitol, a precursor of xylulose 5-phosphate and an intermediary molecule of the pentose phosphate pathway, activates the expression of *ACCase* and *L-PK*, two direct targets of ChREBP, demonstrating that this pathway is functional during pancreatic development. Fourth, xylitol activates beta cell differentiation without affecting acinar cell development. This fits well with the fact that ChREBP is highly enriched in cells of the endocrine pathway. In addition, okadaic acid and calyculin A, two inhibitors of the serine/threonine PP2A, which inhibit ChREBP activity [47, 48], suppress the effects of xylitol on beta cell development. Finally, the production of dnChREBP suppresses the effects of both glucose and GlcNAc, a substrate of the HBP, on beta cell development. Taken together, such data demonstrate that ChREBP tightly regulates beta cell development.

We have recently demonstrated that the HBP mediates, at least in part, the effect of glucose on beta cell differentiation [11]. Specifically, we had proposed that the HBP controlled an *O*-GlcNAcylated factor necessary for proper beta cell differentiation [11]. It is now established that ChREBP is *O*-GlcNAcylated [43, 44]. We propose that glucose controls beta cell differentiation, by promoting ChREBP activity, first by allowing its dephosphorylation, and second through the HBP, which would regulate ChREBP *O*-GlcNAcylation. These two steps, controlled by glucose, would permit efficient beta cell differentiation. One element supporting this hypothesis is the fact that, in pancreases infected with an Ad-dnChREBP, the positive effect of GlcNAc on beta cell differentiation is lost (Fig. 6). Therefore, the effect of glucose on beta cell development, via its metabolism through the HBP, requires an active ChREBP. ChREBP is thus at the crossroads of the different glucose metabolic pathways regulating beta cell differentiation from pancreatic progenitors.

The phenotype of mice deficient in ChREBP was recently analysed [16]. Such mice were glucose-intolerant, but their pancreatic phenotype was not described. In light of our data, it would be interesting to study pancreatic beta cell development in such mice. It will also be interesting to determine whether glucose, through ChREBP activation, controls human beta cell differentiation. Such information would be important in protocols aiming at generating human beta cells from stem cells.

## Electronic supplementary material

Below is the link to the electronic supplementary material.

ESM Fig. 1 (PDF 98 kb)

ESM Fig. 2 (PDF 10712 kb)

ESM Fig. 3 (PDF 122 kb)

ESM Fig. 4 (PDF 171 kb)

ESM Fig. 5 (PDF 118 kb)

## Abbreviations

ACCase: Acetyl-CoA carboxylase
ChREBP: Carbohydrate-responsive element-binding protein
dnChREBP: Dominant-negative carbohydrate-responsive element-binding protein
FAS: Fatty acid synthase
GFP: Green fluorescent protein
GlcNAc: *N*-Acetylglucosamine
HBP: Hexosamine biosynthetic pathway
L-PK: Liver-type pyruvate kinase
NEUROG3: Neurogenin3
PC1/3: Proconvertase 1/3
PDX1: Pancreatic and duodenum homeobox 1
PP2A: Protein phosphatase 2A
