# Supplementary material for: Activation of the transcription factor carbohydrate-responsive element-binding protein by glucose leads to increased pancreatic beta cell differentiation in rats
Source: Diabetologia. 2012 Jul 5;55(10):2713–22. doi: 10.1007/s00125-012-2623-0 (PMC3433661; doi:10.1007/s00125-012-2623-0)
Supplement: Supplementary file 1 — (PDF 98 kb) [file 125_2012_2623_MOESM1_ESM.pdf]

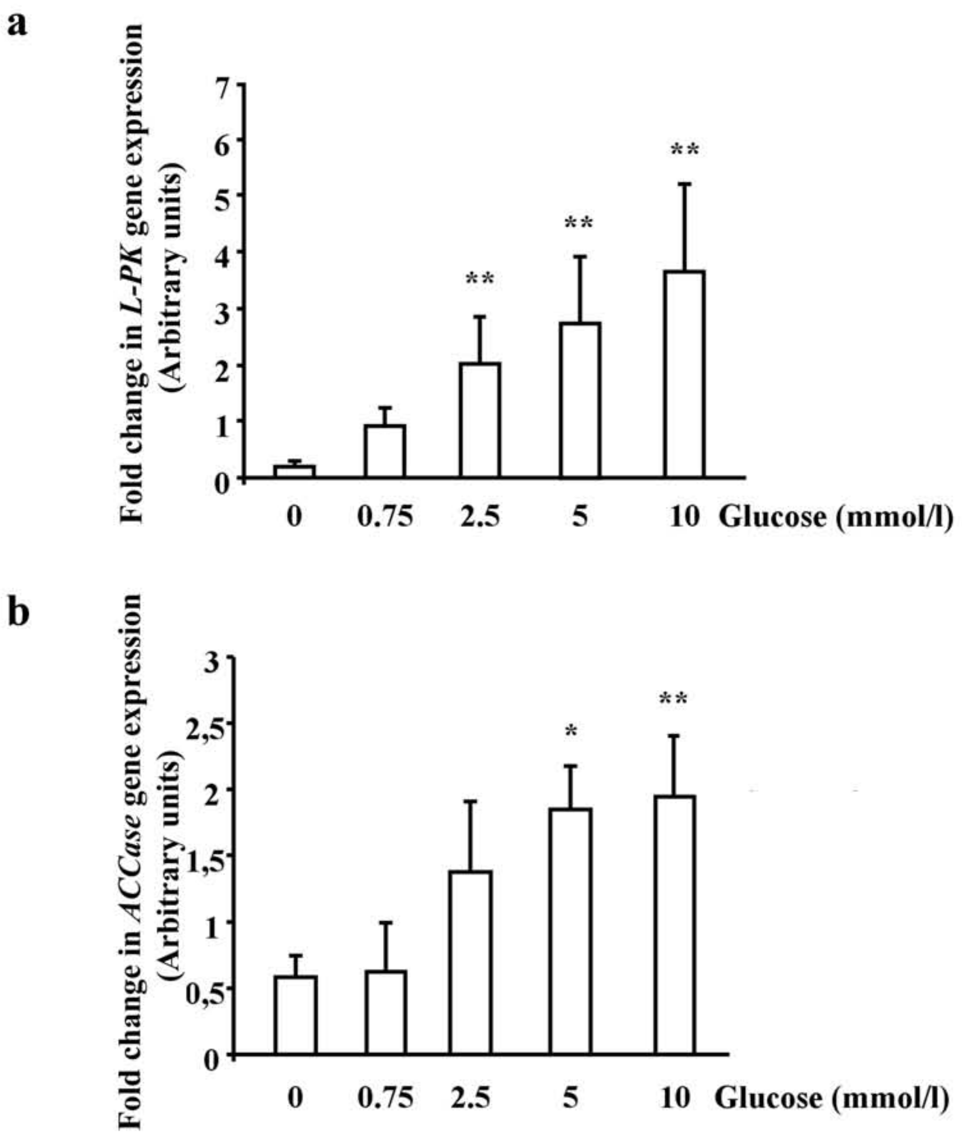

Figure 1: The expression of *L-PK* and *ACCase*, 2 ChREBP targets, is induced in a glucose dose-dependant manner.

Quantification by real-time PCR of *L-PK* and *ACCase* mRNA expression in E13.5 pancreases after 7 days of culture with increasing concentrations of glucose (0, 0.75, 2.5, 5 or 10mmol/l). Values are means + S.E.M. of three independent experiments. *, $p<0.05$; **, $p<0.01$ compared to pancreases cultured in absence of added glucose.
