# Supplementary material for: Activation of the transcription factor carbohydrate-responsive element-binding protein by glucose leads to increased pancreatic beta cell differentiation in rats
Source: Diabetologia. 2012 Jul 5;55(10):2713–22. doi: 10.1007/s00125-012-2623-0 (PMC3433661; doi:10.1007/s00125-012-2623-0)
Supplement: Supplementary file 2 — (PDF 10712 kb) [file 125_2012_2623_MOESM2_ESM.pdf]

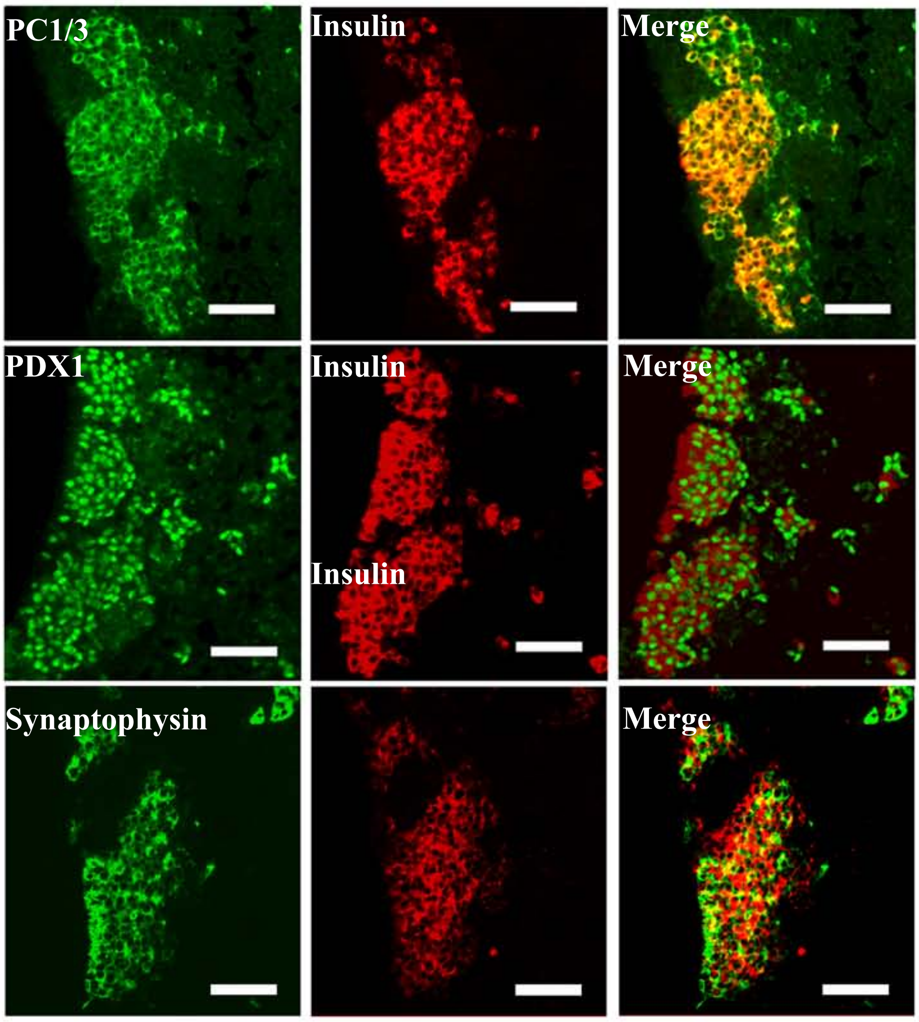

Figure 2: Insulin -positive cells that developed in presence of xylitol produce functional beta cell markers

Immunohistochemical analysis of PC1/3, PDX1 and Synaptophysin, three beta cell markers, after 7 days of culture in presence of 10mmol/l xylitol. PC1/3, PDX1 and synaptophysin were revealed in green (left panels); Insulin was revealed in red (middle panel); the merge picture revealed the colocalization ofthe staining (right panel). Scale bar: 100µm.
