# Supplementary material for: Activation of the transcription factor carbohydrate-responsive element-binding protein by glucose leads to increased pancreatic beta cell differentiation in rats
Source: Diabetologia. 2012 Jul 5;55(10):2713–22. doi: 10.1007/s00125-012-2623-0 (PMC3433661; doi:10.1007/s00125-012-2623-0)
Supplement: Supplementary file 3 — (PDF 122 kb) [file 125_2012_2623_MOESM3_ESM.pdf]

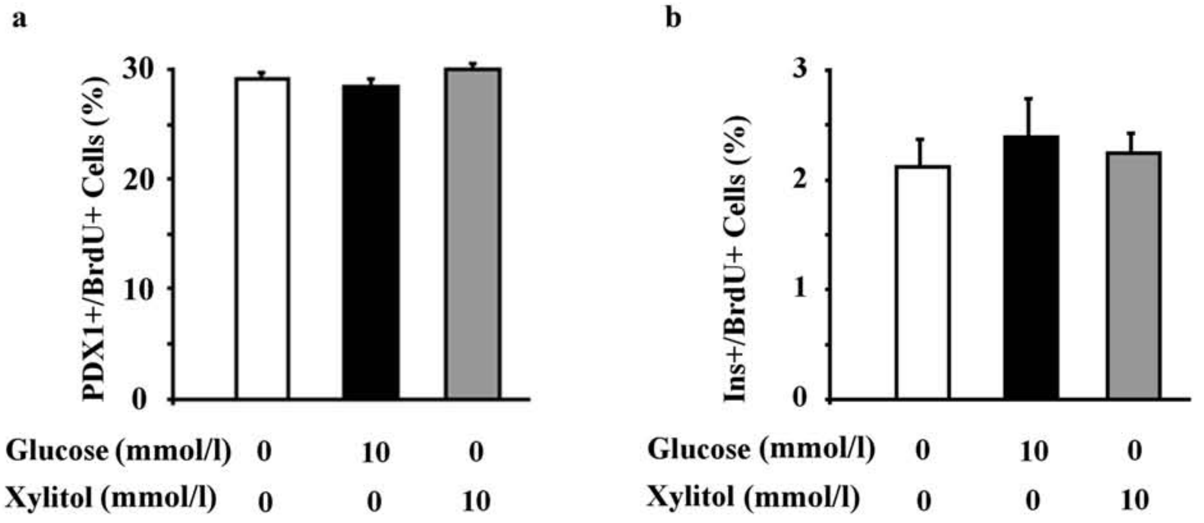

Figure 3: Neither xylitol nor glucose affect progenitor or differentiated beta cells proliferation.

a: E13.5 rat pancreases were grown for 24 h in the presence or in the absence of glucose or xylitol, and pulsed with BrdU during the last hour of culture to ensure labeling of cells in S phase. The tissues were analyzed by immunohistochemistry using anti-PDX1 and anti-BrdU antibodies. The percentage of PDX1-positive cells in S phase was quantified. Values are means ± SEM of three independent experiments.

b: E13.5 rat pancreases were grown for 7 days in the presence or in absence of glucose or xylitol, and pulsed with BrdU during the last hour of culture. The tissues were analyzed by immunohistochemistry using anti-insulin and anti-BrdU antibodies. The percentage of insulin-positive cells in S phase was quantified. Values are means ± SEM of three independent experiments.
