# Supplementary material for: Activation of the transcription factor carbohydrate-responsive element-binding protein by glucose leads to increased pancreatic beta cell differentiation in rats
Source: Diabetologia. 2012 Jul 5;55(10):2713–22. doi: 10.1007/s00125-012-2623-0 (PMC3433661; doi:10.1007/s00125-012-2623-0)
Supplement: Supplementary file 4 — (PDF 171 kb) [file 125_2012_2623_MOESM4_ESM.pdf]

a

b

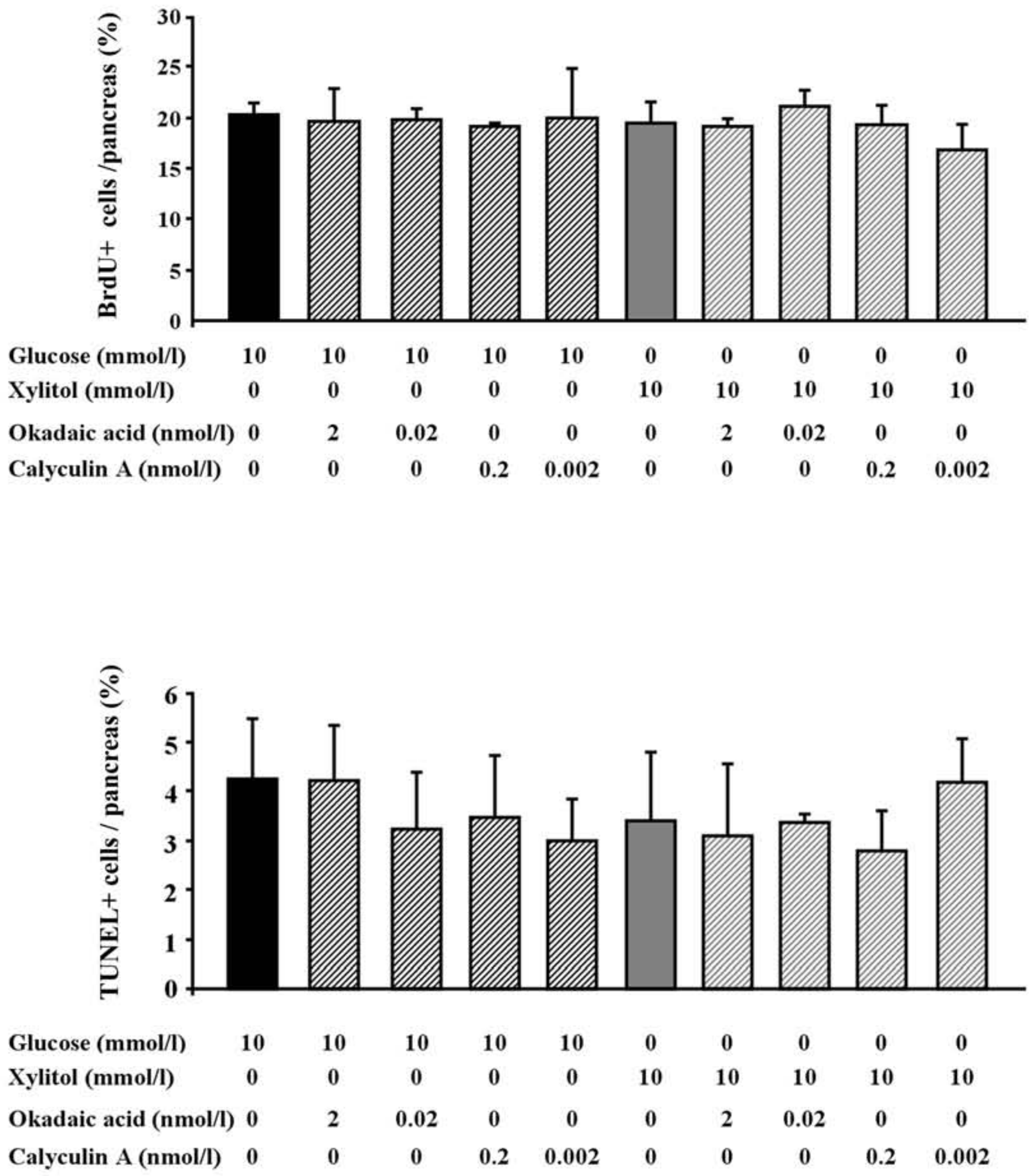

Figure 4: Proliferation and apoptosis were not modified in pancreases cultured in presence of PP2A inhibitors.

a: E13.5 pancreases were grown for 7 days in the presence of glucose or xylitol alone or supplemented with okadaic acid or Calyculin A at the indicated concentrations. Pancreases were pulsed with BrdU during the last hour of culture to ensure labelling of cells in S phase. The tissues were analyzed by immunohistochemistry using anti-BrdU antibody. Nuclei were stained in blue using a Hoechst dye. The percentage of BrdU+ cells per pancreas was quantified.

b: E13.5 pancreases were grown for 7 days in the presence of glucose or xylitol alone or supplemented with okadaic acid or Calyculin A at the indicated concentrations. The tissues were analyzed by immunohistochemistry using *in situ* cell death detection kit from Roche. Nuclei were stained in blue using a Hoechst dye. The percentage of TUNEL+ cells per pancreas was quantified.
