# Supplementary material for: Activation of the transcription factor carbohydrate-responsive element-binding protein by glucose leads to increased pancreatic beta cell differentiation in rats
Source: Diabetologia. 2012 Jul 5;55(10):2713–22. doi: 10.1007/s00125-012-2623-0 (PMC3433661; doi:10.1007/s00125-012-2623-0)
Supplement: Supplementary file 5 — (PDF 118 kb) [file 125_2012_2623_MOESM5_ESM.pdf]

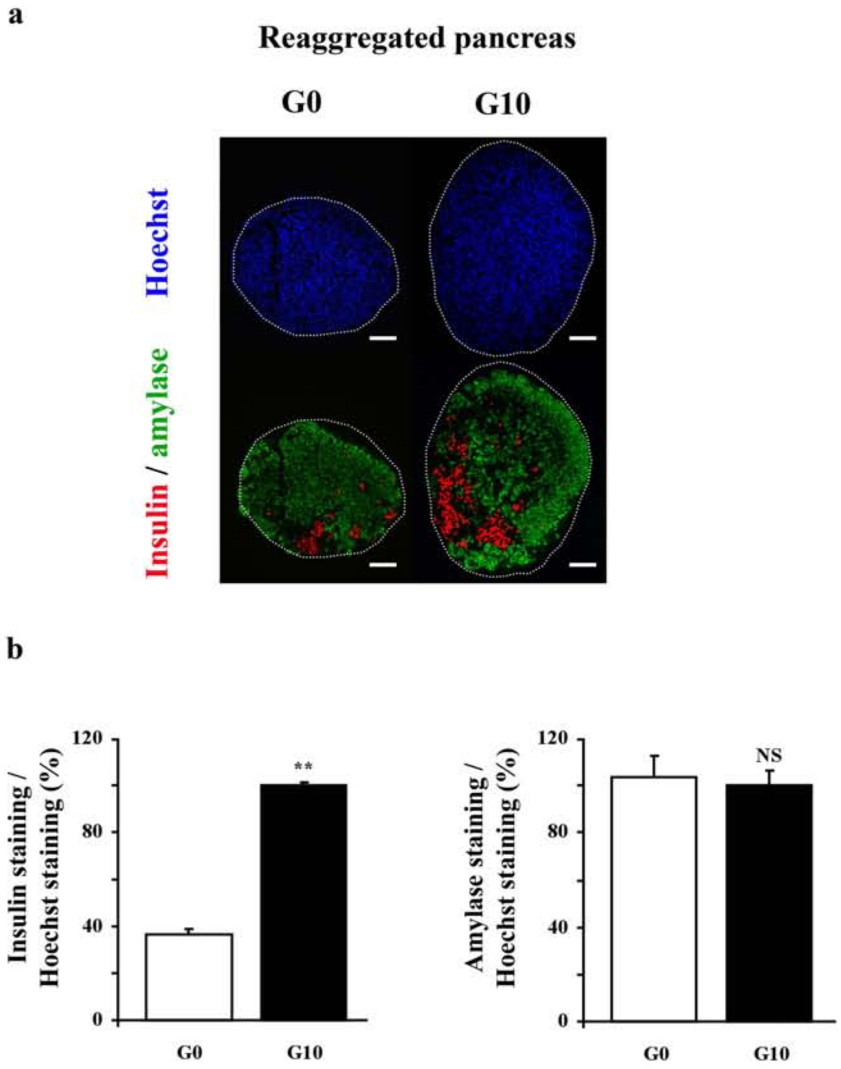

Figure 5: Glucose controls beta cell differentiation in reaggregated pancreases.

a: Immunohistological analysis of reaggregated E13.5 rat pancreases, after 7 days of culture in the absence or in the presence of 10mmol/l added glucose. Insulin was revealed in red, amylase in green, and nuclei were stained in blue using a Hoechst dye. Scale bar, 100µm.

b: Left panel – Quantification of the relative surface area occupied by insulin-positive cells after 7 days of culture in the absence or in the presence of 10mmol/l added glucose. Right panel – Quantification of the relative surface area occupied by amylase-positive cells after 7 days of culture in the absence or in the presence of 10mmol/l added glucose.
